# Epileptogenicity of white matter lesions in cerebral small vessel disease: a systematic review and meta-analysis

**DOI:** 10.1007/s00415-023-11828-6

**Published:** 2023-06-21

**Authors:** Jakob I. Doerrfuss, Jonas M. Hebel, Martin Holtkamp

**Affiliations:** 1 Charité – Universitätsmedizin Berlin, corporate member of Freie Universität Berlin and Humboldt-Universität zu Berlin, Department of Neurology with Experimental Neurology, Campus Benjamin Franklin, Hindenburgdamm 30, 12203 Berlin, Germany; 2 Epilepsy-Center Berlin-Brandenburg, Institute for Diagnostics of Epilepsy, Berlin, Germany

**Keywords:** Epilepsy, Seizures, Stroke, Leukoencephalopathy

## Abstract

**Background:**

The epileptogenic properties of white matter lesions (WML) in cerebral small vessel disease (CSVD) are not yet understood. The aim of our systematic review and meta-analysis was to estimate the association between the extent of WML in CSVD and epilepsy, analyze whether these WML are associated with an increased risk of seizure recurrence, and evaluate if treatment with anti-seizure medication (ASM) is justified in first-seizure patients with WML and no cortical lesions.

**Methods:**

Following a pre-registered study protocol (PROSPERO-ID CRD42023390665), we systematically searched Pubmed and Embase for relevant literature comparing WML load between patients with epilepsy and controls as well as studies on seizure recurrence risk and ASM therapy in the presence vs. absence of WML. We calculated pooled estimates using a random effects model.

**Results:**

Eleven studies comprising 2983 patients were included in our study. Presence of WML (OR 2.14, 95% CI 1.38–3.33) and presence of relevant WML as assessed by visual rating scales (OR 3.96, 95% CI 2.55–6.16) but not WML volume (OR 1.30, 95% CI 0.91–1.85) were significantly associated with seizures. These results stayed robust in sensitivity analyses restricted to studies on patients with late-onset seizures/epilepsy. Only two studies assessed the association between WML and risk of seizure recurrence with conflicting results. Currently, there are no studies on the efficacy of ASM therapy in the presence of WML in CSVD.

**Conclusions:**

This meta-analysis suggests an association between presence of WML in CSVD and seizures. More research is needed addressing the association between WML and risk of seizure recurrence and ASM therapy focusing on a population of patients with a first unprovoked seizure.

**Supplementary Information:**

The online version contains supplementary material available at 10.1007/s00415-023-11828-6.

## Introduction

Stroke and seizures have a strong reciprocal relationship: Cortical stroke is the most common etiology of late-onset epilepsy [1]. Vice versa, late-onset epilepsy of unknown etiology is associated with an up to threefold increased risk of subsequent strokes [2]. Usually, only strokes and other pathologies of cortical localization are considered to be epileptogenic. However, in up to 70% of patients with a first unprovoked seizure, a clear epileptogenic lesion cannot be identified [3]. Even after the diagnosis of epilepsy has been made, in approximately 50% of adult patients, the etiology remains unknown [1, 4]. Yet, in a significant proportion of older patients with a first seizure or epilepsy, white matter lesions (WML) are detected [5–7]. In these patients, WML are mainly caused by cerebral small vessel disease (CSVD) [8, 9]. Their epileptogenic properties are not yet understood. However, in patients with a first unprovoked seizure, it is of utmost clinical importance to know if WML increase the risk of further seizures significantly and thus contribute to fulfill the definition criteria of epilepsy [10]. This would have implications with respect to starting anti-seizure medication (ASM) and concerning the duration of a driving ban. The potential epileptogenic properties of WML are of particular importance in first-seizure patients where the EEG shows no epileptiform discharges. The latter would already indicate epilepsy and in most cases the need for ASM.

There are hypotheses that WML may cause seizures by disruption of subcortical networks, blood–brain barrier dysfunction, impaired cerebral perfusion, and inflammation [11, 12]. Moreover, WML could be a surrogate marker for smaller cortical infarcts that are not detected even by 3 Tesla MRI [13, 14].

To determine whether WML of presumed vascular origin should be considered epileptogenic or not, three questions must be addressed:

1. In patients with a first unprovoked seizure or epilepsy, do the amount and localization of WML of presumed vascular origin differ in comparison to people who never had a seizure?
2. In patients with a first unprovoked seizure or epilepsy, is the presence of WML of presumed vascular origin associated with an increased risk of recurrent seizures as compared to patients without WML?
3. For patients with a first unprovoked seizure and WML of presumed vascular origin with no cortical structural lesions in MRI, does treatment with ASM reduce the risk of seizure recurrence compared to patients with no pharmacological treatment?

This systematic review and meta-analysis aim to provide an overview of the current evidence regarding these very questions.

## Methods

This systematic review and meta-analysis follow the 2020 Preferred Reporting Items for Systematic Reviews and Meta-Analysis (PRISMA) guidelines [15]. The review protocol was registered in the PROSPERO database for systematic reviews prior to the commencement of the literature search (registration number CRD42023390665).

### Search strategy

We systematically searched the Embase and MedLine databases using OVID on February 3, 2023. The full search strategy can be accessed in the supplemental material (Table E1, online only). The literature search included studies published from January 2003 to February 2023, and was restricted to studies in English language. Editorials, case reports, comments, and review articles were not included in this systematic review and meta-analysis. As several research questions were epidemiological, we considered observational studies as eligible. To answer our research questions, all studies needed a control group consisting of patients without seizures/epilepsy (research question 1), without WML (research question 2), or without pharmacological treatment (research question 3).

Duplicates in the search results were removed through a combination of automated and manual methods using EndNote. Two authors (JID and JMH) independently screened titles and abstracts. The same authors then assessed the full-texts of potentially eligible studies to decide on inclusion. Discrepancies in the assessment of eligibility were resolved by consensus between the authors.

### Data extraction and synthesis

In a next step, we extracted data on study characteristics, patient population, exposure, and outcome using a pre-specified template. The following data were extracted: author, year of publication, study design, sample size, size and properties of case groups and control groups, mean age, sex, quantification method of WML, imaging modality, outcome assessment, and outcomes (presence of WML, lesion load and volume with mean and standard deviation, seizure recurrence rate, effect estimates and 95% confidence intervals (CI) where applicable).

### Assessment of risk of bias

Risk of bias for each study was assessed independently by two of the authors (JID and JMH) using the case–control subscale of the Newcastle–Ottawa Scale (NOS) for assessing the quality of nonrandomized studies in meta-analyses [16]. This score ranges from 0 to 9 with a score of ≥ 7 indicating a study of high methodical quality. The NOS comprises the domains ‘selection’, ‘comparability’ and ‘outcome’. In the domain ‘comparability’, a maximum of two points can be awarded. One point is given if the study controls for the most important risk factor as selected by the raters. Another point is awarded if the study controls for any additional factor. We considered the cardiovascular risk profile to be the most important factor to control for. Discrepancies in the assessment of bias were resolved through consensus. The authors then assessed the overall quality of evidence for each research question using the Grading of Recommendations, Assessment, Development, and Evaluation (GRADE) scale. The GRADE scale is a 4-point rating scale, ranging from *‘very low’* (the true effect is probably markedly different from the estimated effect) to ‘*high’* (the authors have a lot of confidence that the true effect is similar to the estimated effect). Observational studies are primarily given a certainty rating of ‘low’ with several factors being able to reduce or increase the certainty of evidence [17].

### Missing data

To obtain relevant missing data, we contacted the corresponding authors of six studies through email. Authors of three studies sent additional data.

### Statistical analysis

In our meta-analysis, we calculated summary Odds Ratio (OR) and 95% Confidence Intervals (CI) for the association of seizures or epilepsy and WML as measured by different WML quantification methods.

For the presence of any WML and the presence of relevant WML (Fazekas scale score > 1 or ARWMC scale score > 2), we extracted frequencies in the epilepsy/seizure groups and the control groups from each study and then performed a random effects meta-analysis for binary outcomes.

For the association between WML volume and epilepsy, we extracted effect estimates from studies if they were provided. If these were not given in the studies, we requested original data from the corresponding authors and calculated adjusted effects estimates for each study with z-scores of WML volume (adjustment for age and sex; inclusion method:enter, *p* < 0.05 [p in], *p* < 0.1 [*p* out], iteration 20, and constant included). We then pooled the log-transformed OR and 95% CIs using a random effects model.

To assess heterogeneity, Cochrane Q-statistics were calculated and quantified using the *I*^2^ values. We considered an *I*^2^ value > 50% as significant heterogeneity. We assessed publication bias by visual exploration of Funnel plots and Egger’s test. All statistics were performed using SPSS Version 28 (IBM, Chicago, USA).

## Results

### Literature search and study characteristics

**Fig. 1:**
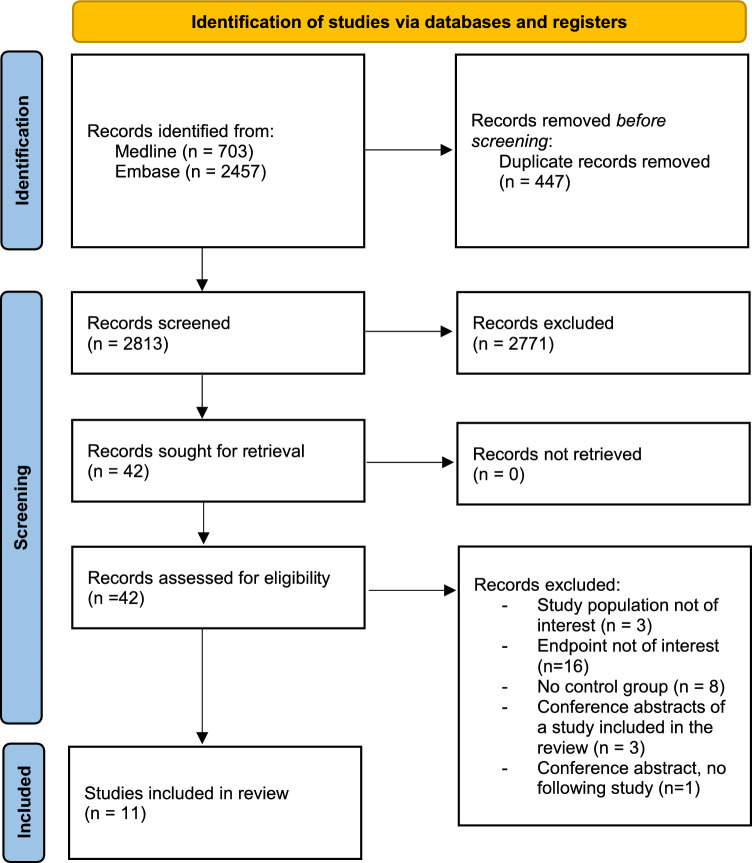
PRISMA Flow chart of inclusion of studies

A total of 2813 records were screened of which 11 studies were finally included in this systematic review. Figure 1 shows the PRISMA flow chart.

The included studies were published between 2006 and 2022. Ten studies reported WML load of patients with first unprovoked seizure and/or epilepsy as compared to control groups (research question 1) [5–7, 18–25]. Two studies compared risk of seizure recurrence in patients with and without WML (research question 2) [18, 21]. We could not identify any study on treatment with ASM in patients with CSVD-related WML (research question 3). The characteristics of all included studies including imaging modality, properties of the control groups, and WML quantification method are summarized in Table 1. The studies included in this systematic review comprised a total of 2983 patients; 862 cases and 2121 controls. According to our quality assessment, three studies were of high methodological quality (Table 1, and for more detail: Table E2, online only).

**Table 1:** Study characteristics

Author	Year	Study design		Number of participants	Number of cases	Number of controls	Mean age cases (years)	Mean age controls (years)	Properties of control group	Imaging modality		WML quantification method	NOS
		Retrospective case–control	Prospective case–control							MRI	CT		
Abraira	2019	✓		100	31	41/26^a^	70.9	73.2/68.0	-Control group I: patients with transient ischemic attack or lacunar infarction and no cortical infarcts -Control group II: healthy controls	✓		Fazekas scale ranging from 0–3, including deep WML and periventricular WML	5
De Reuck	2007	✓		242	37	205	nr	nr	Patients with acute lacunar stroke	✓	✓	Degree of white matter changes, not further specified	3
Hanby	2015		✓	31	16	15	67.6	65.1	Healthy controls	✓		WML volume	6
Jansen	2008		✓	49	33	16	43	40	Healthy controls	✓		WML volume	5
Johnson	2019	✓		1,526/ 1,404^b^	97/ 28^b^	1,429/ 1,372^b^	78.3	77.0	Participants of a community-based study on atherosclerosis risk without seizures	✓		WML volume WML grade score (0–9)	8
Mao	2016	✓		240	217	23	42	36	Healthy controls	✓		ARWMC scale ranging from 0–30	8
Maxwell	2013	✓		210	105	105	72.7	72.4	Healthy controls	✓	✓	Fazekas scale ranging from 0–3, not including periventricular WML	4
Stösser	2019	✓		236	118	118	82 (median)	79 (median)	Patients with transient ischemic attack	✓		Fazekas scale for periventricular and deep lesions and global (modified) score, each ranging from 0–3^c^ ARWMC scale ranging from 0–3 for each region	5
Tartara	2022	✓		162	87	75	nr	nr	Patients without leukoaraiosis	✓	✓	Fazekas scale ranging from 0–3, not further defined	6
Turon	2021		✓	54	27	27	74.2	72.1	Patients with CSVD (subcortical microbleeds, Fazekas score ≥ 1 or ≥ 1 lacunar infarct)	✓		(a) Fazekas scale for deep WML, dichotomized as Fazekas < 2 vs. Fazekas ≥ 2 and Fazekas scale for periventricular WML (b) 4-point CSVD burden score including WML, lacunar infarcts, microbleeds, and enlarged perivascular spaces	5
Uslu	2021		✓	135	94	41	30.5	33.1	Healthy controls	✓		“global” ARWMC scale, defined as score of the region with the most severe WML ranging from 0–3	7

The NOS scale ranges from 0 to 9 with a higher score indicating a superior quality. A score of ≥ 7 indicates a study of high methodological quality

*nr* not reported, *na* not applicable, *ARWMC* age-related white matter changes, *WML* white matter lesions, *NOS* Newcastle–Ottawa Scale, *CSVD* cerebral small vessel disease, *CT* computed tomography, *MRI* magnetic resonance imaging

^a^Two control groups, the control group “healthy individuals” was included in the meta-analysis

^b^Two overlapping study populations, the second study population for which a white matter hyperintensity volume was provided was included in the meta-analysis

^c^The global score was used in the meta-analysis

### Research question 1: In patients with a first unprovoked seizure or epilepsy, do the amount and localization of WML of presumed vascular origin differ in comparison to people who never had a seizure?

Studies assessing WML load in cases and controls reported either (a) presence of any WML and/or (b) WML load as assessed by a visual rating scale and/or (c) WML volume. One study reported a not further specified degree of white matter changes and was therefore not included in any of the following meta-analyses [24]. A detailed description of the properties of the epilepsy groups as well as individual results regarding lesion load for each study can be found in the supplementary material (Table E3, online only).

#### Meta-analysis on presence of WML

Six studies reported presence of WML in 600 cases and 329 controls [5, 6, 18, 20, 23, 25]. Of these studies, four covered only patients with epilepsy [5, 18, 23, 25], one study included only patients with respective first seizures [6], and one study investigated patients with either a first seizure or epilepsy [20]. None of the four studies focusing on patients with epilepsy stated whether patients had new-onset epilepsy or already established epilepsy. One study had two control groups: patients with a transient ischemic attack or lacunar infarction and one control group with healthy controls. Only the second control group was used for the current meta-analysis to facilitate comparability [25].

**Fig. 2:**
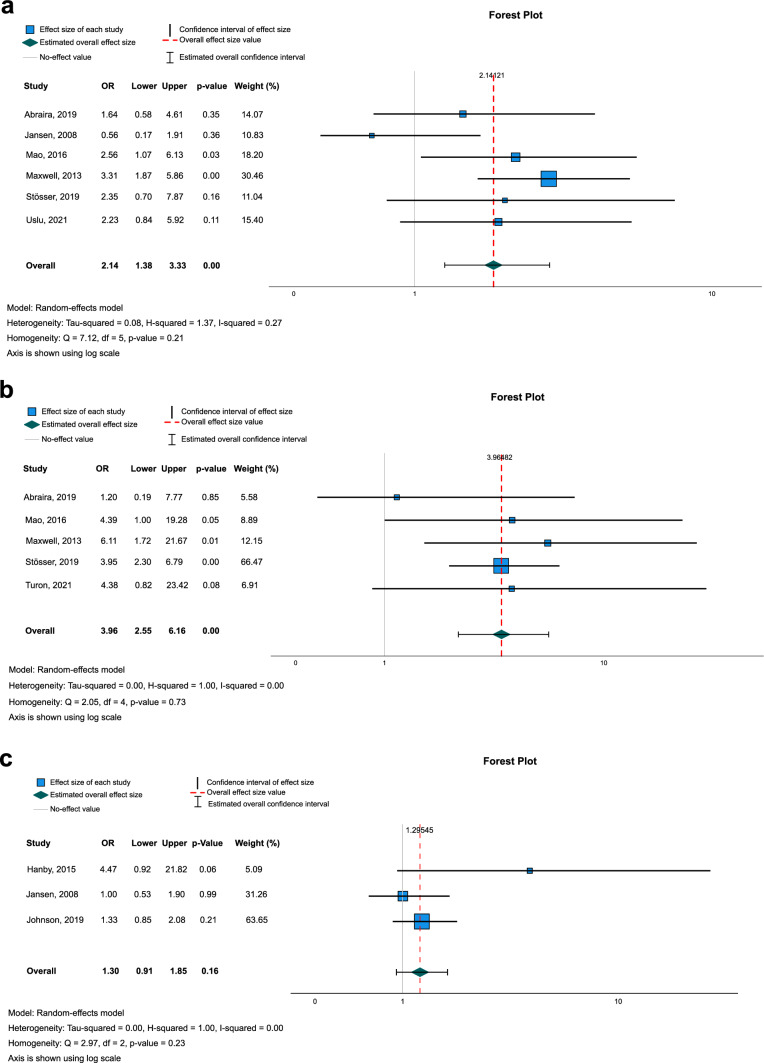
Meta-analysis on the association between epilepsy and **a** presence of WML, **b** relevant WML as assessed by visual rating scales, and **c** WML volume

Patients with a first unprovoked seizure or epilepsy had WML more frequently than the control group (OR 2.14, 95% CI 1.38–3.33, *I*^2^ = 0.27, Fig. 2a). This association was more pronounced in a sensitivity analysis including only studies on late-onset seizures in patients aged ≥ 60 years (OR 2.74, 95% CI 1.72–4.34, *I*^2^ = 0.00) [6, 20, 25]. The results stayed robust in a sensitivity analysis excluding the single study that only included patients with respective first seizures and in which the control group did not consist of healthy controls (OR 2.05, 95% CI 1.22–3.46, *I*^2^ = 0.41) [6].

**Fig. 3:**
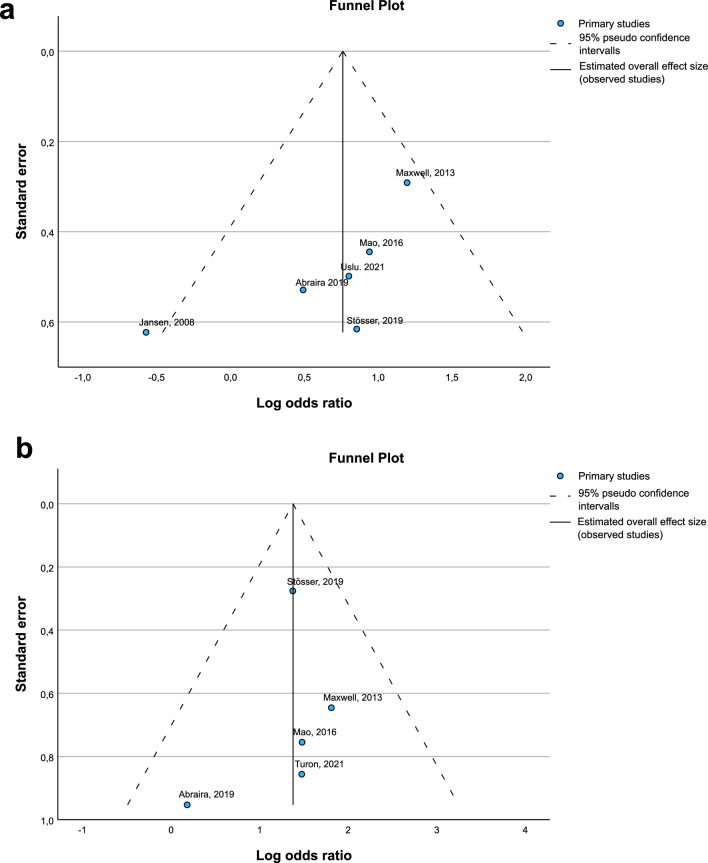
Funnel plots. This figure shows funnel plots for the association between epilepsy and **a** presence of WML, and **b** relevant WML as assessed by visual rating scales. Due to the small number of studies on WML volume (*n* = 3), we did not calculate funnel plots for the association between epilepsy and WML volume

Visual analysis of the funnel plot for the primary analysis as well as Egger’s test (*p* = 0.11) showed no significant asymmetry and was therefore not indicative of a relevant publication bias (Fig. 3a). According to the GRADE certainty ratings, we rate the certainty of evidence as *‘low’.*

#### Meta-analysis on relevant WML load as measured by visual rating scales

A total of five studies reported the extent of WML as measured by visual rating scales [5, 6, 19, 20, 25]. These studies comprised 500 cases and 299 controls. Two studies included only patients with epilepsy [5, 25], one study investigated only patients with first seizures [6], and two studies included patients with both first seizures and epilepsy [19, 20].

For quantification of WML, all studies used variants of the Fazekas and Wahlund scales, although the application of these scales differed [26, 27]. We considered a score of > 1 on the Fazekas scale (scale range 0–3) and a score of > 2 Wahlund scale (scale range 0–30) as ‘relevant’ WML load. Its presence was associated with seizure(s) (OR 3.96, 95% CI 2.55–6.16, *I*^2^ = 0.00 Fig. 2b). A sensitivity analysis excluding the single study that was not on late-onset seizures/epilepsy and used the Wahlund scale instead of the Fazekas scale [5] showed similar results (OR 3.93, 95% CI 2.47–6.23, *I*^2^ = 0.00). The results also remained largely unchanged in a sensitivity analysis restricted to studies using a control group of healthy participants (OR 3.89, 95% CI 3.89, 95% CI 1.65–9.15, *I*^2^ = 0.00) [5, 20, 25].

Again, visual inspection of the funnel plot and Egger’s Test (*p* = 0.77) were not evident of a relevant publication bias (Fig. 3b).

For the association of a relevant WML load as measured by visual rating scales and seizure(s), we award a GRADE certainty rating of *‘low’*.

#### Meta-analysis on WML volume

Three studies (77 patients, 1403 controls) assessed WML volume in patients with and without epilepsy [7, 22, 23]. None of these studies assessed patients with first seizures or new-onset epilepsy. One study was a retrospective case–control study, which analyzed the association of the degree and volume of WML earlier in life, as measured in the Arteriosclerosis Risk in Communities (ARIC) study, with epilepsy that was diagnosed later in life [22].

Mean WML volume in the three studies ranged from 160 to 21,800 mm^3^ in the epilepsy group and 203 to 17,500 mm^3^ in the control group. All WML volumes were confirmed (and in one study corrected) by the corresponding authors. The numbers given here are the corrected values.

Due to extensive heterogeneity of WML volume between different studies, a meta-analysis of mean differences was not deemed appropriate. We instead analyzed whether WML volume was independently associated with presence of epilepsy. For this analysis, we used the reported adjusted OR from one study [22], and with original data provided by the authors, calculated OR for z-scores of WML volume adjusting for age and sex for the two other studies [7, 23]. The following meta-analysis showed no association between WML volume and presence of epilepsy (OR 1.30, 95% CI 0.91–1.85, *I*^2^ = 0.00, Fig. 2c). In a sensitivity analysis excluding the one study in which MRI was performed before onset of epilepsy and in which the controls were not necessarily healthy, the results remained without a statistically significant difference (OR 1.76, 95% CI 0.43–7.28, *I*^2^ = 0.66) [22]. The same was true for another sensitivity analysis excluding the one study that was not on late-onset epilepsy (OR 1.90, 95% CI 0.64–5.62 *I*^2^ = 0.52) [23]. Because of the small number of studies, we did not test for funnel plot asymmetry.

Overall, with a GRADE certainty rating of *‘very low’* due to inconsistency of results, there was no association between WML volume and epilepsy.

### Research question 2: In patients with a first unprovoked seizure or epilepsy, is the presence of WML of presumed vascular origin associated with an increased risk of recurrent seizures as compared to patients without WML?

We identified two studies analyzing the association between risk of seizure recurrence and WML [18, 21]. These studies included a total of 256 patients with epilepsy of whom 113 had and 143 did not have WML. The characteristics of these studies are summarized in Table 1.

The first study [21] focused on patients with late-onset epilepsy of unknown or structural etiology. Presence of WML was independently associated with an elevated risk of seizure recurrence (RR 1.76, 95% CI 1.01–3.06). The study showed no association between severity of WML and seizure frequency.

The main focus of the second study [18] was to compare lesion load between patients with epilepsy (age ≥ 18 years, exclusion of patients with cardiovascular risk factors) and a control group; therefore, this study is also part of the meta-analysis on presence of WML. In the epilepsy group, presence of WML was not associated with seizure frequency (*p* = 0.444). However, the assessment of seizure frequency in this study was not further specified, and seizure frequencies in the case and control groups were not reported. Thus, it was impossible to conduct a meta-analysis of these two studies. Additional information on these two studies can be accessed in the supplemental material (Table E4, online only).

Overall, there is little and conflicting evidence on the association between WML and seizure frequency. We rate the GRADE certainty of this association as *‘very low’* due to inconsistency of the results.

### Research question 3: For patients with a first unprovoked seizure and WML of presumed vascular origin with no cortical structural lesions in MRI, does treatment with ASM reduce the risk of seizure recurrence compared to patients with no pharmacological treatment?

In our systematic review, no studies on the treatment of patients with WML of presumed vascular origin and first unprovoked seizure without cortical structural lesions could be identified.

## Discussion

This is the first systematic review and meta-analysis on the association of WML of presumed vascular origin with seizures or epilepsy. Gathering data from 11 studies comprising almost 3000 participants, we analyzed the current evidence on the association of WML with the presence of seizures, rate of seizure recurrence, and necessity of ASM treatment.

### Association between WML and presence of epilepsy and seizures

There was a statistically significant association between (a) seizure or epilepsy and presence of WML, (b) seizure or epilepsy and presence of relevant WML as measured by a visual rating scale, but no association between (c) WML volume and epilepsy.

Given the current evidence, it is difficult to determine, whether this discrepancy is due to methodological or pathophysiological reasons. It is important to mention that there are significant differences in the design of the three studies on WML volume and epilepsy [7, 22, 23]. The only study to show a statistically significant difference in WML volume load was on patients with late-onset epilepsy > 50 years [7]. One study had a much younger population (mean age in epilepsy group: 43 years) [23]. The third study did not assess lesion load at onset of epilepsy. Instead, the authors analyzed MRIs that were performed before the occurrence of the respective first seizures, with the time from MRI to first seizure not being reported [22]. After excluding this study from the meta-analysis, the results regarding association between WML volume and epilepsy remained without statistically significant difference. The differences in mean WML volume between these three studies are striking. To our understanding, this could only partly be explained by differences in methods of WML segmentation. Therefore, we confirmed the WML volumes with the corresponding authors of all three studies to ensure the plausibility of the data. This led to the correction of WML volumes in one study. Still, in the epilepsy group, a 16-fold difference in mean WML volume between the study with the highest and the lowest WML volume remained [7, 22]. Reassuringly, all reported WML volumes are within the range of what has been described previously [28, 29]. Therefore, it might be reasonable to assume that in fact, significant WML as detected by a visual rating scale are more relevant with respect to epilepsy than WML volumes. The Fazekas scale as applied by most authors in the studies included in this meta-analysis focuses on deep WML located > 13 mm from the ventricular surface [27, 30]. In contrast, a WML volumetry takes into account WML in the entire brain including periventricular and juxtacortical WML. It is generally accepted that deep WML differ from WML in other locations like periventricular and juxtacortical regarding histopathology and clinical relevance: Deep and confluent white matter alterations are more likely of ischemic origin and are more often associated with clinical symptoms than periventricular and juxtacortical WML [8, 31, 32]. This distinction is better represented by visual scores than by a global quantitative measurement. Results of one study in which presence of deep and periventricular WML was analyzed separately are in line with this notion: Deep WML were more common in the epilepsy group, while periventricular WML were more common in the control group [19]. In a study comparing first-seizure patients to patients with transient ischemic attacks, both periventricular and deep WML were more present in the seizure group [6]. The same study also showed a statistically significant higher prevalence of juxtacortical lesions in the first-seizure group as compared to the control group (80.5% vs 22%, *p* < 0.001). Finally, in a study that was considered for eligibility in the systematic review but was excluded due to lack of a control group, in patients with epilepsy, WML mainly affected the temporal lobe [13].

Importantly, there is also heterogeneity in the studies on presence of (relevant) WML as assessed by visual rating scales. This is mainly represented in (a) patient populations and (b) differences in visual WML scoring. Regarding patient populations of these studies, four included patients with late-onset seizures [6, 19, 20, 25] while two had no age restrictions [5, 18], and one study did not report the age of its participants [24]. There were also significant differences within comorbidities of the patient populations, especially with regard to cardiovascular risk factors [18, 19]. Another challenge in comparing studies were differences in WML scoring: The most widely used scale for WML was proposed by Fazekas et al. [27] more than 30 years ago. In this scale, both periventricular and deep WML are scored separately on a 4-point rating scale ranging from 0 to 3. To assess CSVD, usually only deep WML are considered [27]. The so-called age-related white matter changes (ARWMC) or Wahlund scale is an updated version of the Fazekas scale. Again, WML are scored on a 4-point rating scale with slight alterations in scoring. More importantly, in this scale, five different brain regions are scored for each hemisphere separately. Thus, a maximum of 30 points theoretically can be given [26].

Even though most studies included in this meta-analysis refer to the paper introducing the Wahlund scale, they in fact mostly provide a “global” or “deep” WML score ranging from 0 to 3, which is more in line with the Fazekas scale in its original form. Out of the seven studies using a visual rating scale, none applied the exact same scoring method. A standardization of WML rating scales to be used in clinical studies would be highly desirable.

So far, this review has interpreted the assumed association between WML and seizures as unidirectional in the sense that WML might cause seizures. However, also the inverse relationship is worthy of discussion. This point is of particular importance, as none of the studies included in this meta-analysis that focused on epilepsy (as opposed to first-seizure patients), stated that it was on new-onset epilepsy patients. Therefore, we do not have certainty on the lesion load at the onset of epilepsy. Thus, given the current data, we cannot exclude that a significant proportion of WML developed after the diagnosis of epilepsy. From a pathophysiological perspective, it seems unlikely that seizures could directly cause WML [33]. Yet, it is possible that epilepsy indirectly causes WML through ASM therapy. Interestingly, in one study included in this review, WML load was highest in patients receiving enzyme-inducing ASM as compared to patients with non-enzyme-inducing ASM and controls without epilepsy [5]. This was interpreted to be caused by negative effects of enzyme-inducing ASM on cardiovascular risk factors [34].

To answer our research questions, only studies with an appropriate control group were eligible for inclusion. It is, however, important to mention that there was heterogeneity in the properties of the control groups in the studies included in this review. We therefore performed sensitivity analyses, including only studies where the control group consisted of healthy participants. Reassuringly, our results stayed robust for all outcome parameters.

### Association between WML and risk of seizure recurrence

We identified two studies on seizure recurrence risk in patients with epilepsy with conflicting results. One study focused on patients with late-onset epilepsy of unknown or structural etiology. Here, presence of WML was independently associated with an elevated risk of seizure recurrence [21]. The other study did not show any statistically significant difference in seizure frequency between patients with and without WML [18]. It is noteworthy, that this study has extensive exclusion criteria: In essence, patients with cardiovascular risk factors, such as hypertension and diabetes mellitus, were not eligible for inclusion [18]. To us, excluding these patients seems counter-intuitive, especially when considering the presumed pathomechanisms behind the suspected epileptogenic properties of WML, namely the hypothesis of WML being a surrogate parameter of cortical microinfarcts [13, 14]. Moreover, it has been demonstrated that cardiovascular risk factors are higher in late-onset epilepsy patients than in controls [35]. It is therefore plausible that this study did underestimate the effect of WML on seizure recurrence.

Assessing the influence of WML on the risk of seizure recurrence is an important milestone in evaluating their epileptogenicity. Both studies identified in this review were on patients with established diagnoses of epilepsy. As these patients are usually treated with ASM, a more desirable study population would be patients with first seizures not yet treated. Only this population would allow assessing the influence of WML on the rate of seizure recurrence without the risk of bias by ASM.

### ASM treatment of patients with WML in the absence of cortical lesions

Ultimately, all aspects discussed above lead to the final, clinically most relevant question, whether ASM treatment is justified or even necessary in patients with a first seizure at the presence of WML, even if there are no cortical lesions in the MRI and the EEG is without epileptiform discharges. Our systematic review did not show any prospective studies on ASM treatment in patients with WML with regard to seizure outcome.

### Strengths, limitations, and scope of this review

The strength of this review lies in its rigorous adherence to the PRISMA guidelines and its meta-analysis using original patient data where possible.

There are limitations to consider. We have already addressed the significant heterogeneity between studies on WML lesion load. Through several sensitivity analyses, we aimed to diminish its effects, but even so, the certainty of evidence remains impaired. This is especially relevant for the fact that all studies included in this review and meta-analysis were observational studies. Thus, the overall quality of evidence included in this review has to be considered as low. In our opinion, a major limitation in the design of most included studies was the lack of adjustment for cardiovascular risk factors (see Table E2, online only).

Initially, we had set out to compare WML load in first-seizure patients and epilepsy patients separately. However, only one study was identified focusing on first-seizure patients [6]. In two studies, a small number of patients with a single seizure were also part of the respective “epilepsy group”, and neither of them performed a separate analysis [20, 24]. A sensitivity analysis without the study only on first-seizure patients did not significantly alter the results of our meta-analysis.

This review explicitly focuses on WML of presumed vascular origin. It is important to mention that CSVD is more than WML. Other signs of CSVD include lacunar infarcts, enlarged perivascular spaces, micro-bleeds, and brain atrophy [8]. These signs were assessed as secondary outcomes by some studies included in this review [6, 7, 19, 20, 22, 25]. To summarize, it was mainly shown that epilepsy was associated with reduced cortical volume and hippocampal or temporal atrophy [6, 7, 22, 25]. This seems plausible from a pathophysiological viewpoint. Also, CSVD in general (including lacunar infarcts, perivascular spaces, and micro-bleeds along with WML) was more prevalent in patients with epilepsy than in controls [19, 20]. The topic of this review on WML of presumed vascular origin also implicates that we did not look into microstructural alterations of white matter as detected in diffusion tensor imaging studies, which were reviewed elsewhere [36].

## Conclusions

Our findings suggest that WML are more frequent in patients with epilepsy than in controls. It seems reasonable to assume that WML of presumed vascular origin contribute to epileptogenesis in patients with late-onset epilepsy. This is especially true for deep WML. As of now, there has not been shown a clear association between WML volume and epilepsy. Currently, evidence is insufficient to answer the questions whether WML are associated with an increased risk of seizure recurrence, and thus, if patients with a first seizure and WML should receive ASM, even in the absence of cortical lesions. In our opinion, these two aspects should be addressed in further studies, ideally focusing on a first-seizure patient population, with an emphasis on alterations of the deep white matter.

### Supplementary Information

Below is the link to the electronic supplementary material.

Supplementary file1 (PDF 456 KB)

## Data Availability

Data are available on request from the corresponding author.

## References

[1] Tanaka A, Akamatsu N, Shouzaki T, Toyota T, Yamano M, Nakagawa M, Tsuji S (2013). Clinical characteristics and treatment responses in new-onset epilepsy in the elderly. Seizure.

[2] Cleary P, Shorvon S, Tallis R (2004). Late-onset seizures as a predictor of subsequent stroke. Lancet.

[3] Ho K, Lawn N, Bynevelt M, Lee J, Dunne J (2013). Neuroimaging of first-ever seizure: contribution of MRI if CT is normal. Neurol Clin Pract.

[4] Doerrfuss JI, Kowski AB, Holtkamp M (2021). Etiology-specific response to antiseizure medication in focal epilepsy. Epilepsia.

[5] Mao YT, Goh E, Churilov L, McIntosh A, Ren YF, O'Brien TJ, Davis S, Dong Q, Yan B, Kwan P (2016). White matter hyperintensities on brain magnetic resonance imaging in people with epilepsy: a hospital-based study. CNS Neurosci Ther.

[6] Stosser S, Bockler S, Ludolph AC, Kassubek J, Neugebauer H (2019). Juxtacortical lesions are associated with seizures in cerebral small vessel disease. J Neurol.

[7] Hanby MF, Al-Bachari S, Makin F, Vidyasagar R, Parkes LM, Emsley HC (2015). Structural and physiological MRI correlates of occult cerebrovascular disease in late-onset epilepsy. Neuroimage Clin.

[8] Wardlaw JM, Smith EE, Biessels GJ, Cordonnier C, Fazekas F, Frayne R, Lindley RI, O'Brien JT, Barkhof F, Benavente OR (2013). Neuroimaging standards for research into small vessel disease and its contribution to ageing and neurodegeneration. Lancet Neurol.

[9] Erten-Lyons D, Woltjer R, Kaye J, Mattek N, Dodge HH, Green S, Tran H, Howieson DB, Wild K, Silbert LC (2013). Neuropathologic basis of white matter hyperintensity accumulation with advanced age. Neurology.

[10] Fisher RS, Acevedo C, Arzimanoglou A, Bogacz A, Cross JH, Elger CE, Engel J, Forsgren L, French JA, Glynn M (2014). ILAE official report: a practical clinical definition of epilepsy. Epilepsia.

[11] Gibson LM, Allan SM, Parkes LM, Emsley HC (2011). Occult cerebrovascular disease and late-onset epilepsy: could loss of neurovascular unit integrity be a viable model?. Cardiovasc Psychiatry Neurol.

[12] Gibson LM, Hanby MF, Al-Bachari SM, Parkes LM, Allan SM, Emsley HC (2014). Late-onset epilepsy and occult cerebrovascular disease. J Cereb Blood Flow Metab.

[13] Gasparini S, Ferlazzo E, Beghi E, Sofia V, Mumoli L, Labate A, Cianci V, Fatuzzo D, Bellavia MA, Arcudi L (2015). Epilepsy associated with Leukoaraiosis mainly affects temporal lobe: a casual or causal relationship?. Epilepsy Res.

[14] van Veluw SJ, Zwanenburg JJ, Engelen-Lee J, Spliet WG, Hendrikse J, Luijten PR, Biessels GJ (2013). In vivo detection of cerebral cortical microinfarcts with high-resolution 7T MRI. J Cereb Blood Flow Metab.

[15] Page MJ, McKenzie JE, Bossuyt PM, Boutron I, Hoffmann TC, Mulrow CD, Shamseer L, Tetzlaff JM, Akl EA, Brennan SE (2021). The PRISMA 2020 statement: an updated guideline for reporting systematic reviews. Rev Esp Cardiol (Engl Ed).

[16] Wells G, Shea B, O'Connell D, Peterson J, Welch V, Losos M, Tugwell P (2014) The Newcastle-Ottawa Scale (NOS) for assessing the quality of nonrandomised studies in meta-analyses. https://www.ohri.ca/programs/clinical_epidemiology/oxford.asp. Accessed 23 March 2023

[17] GRADE Working Group. Overview of the GRADE approach. https://www.gradeworkinggroup.org/. Accessed 24 March 2023

[18] Uslu FI, Cetintas E, Yurtseven I, Alkan A, Kolukisa M (2021). Relationship of white matter hyperintensities with clinical features of seizures in patients with epilepsy. Arq Neuropsiquiatr.

[19] Turon M, Jimenez-Balado J, Abraira L, Fonseca E, Quintana M, Toledo M, Delgado P, Maisterra O, Salas-Puig X, Alvarez-Sabin J (2021). Effect of late-onset epilepsy on cognitive functioning in patients with small vessel disease. Epilepsy Behav.

[20] Maxwell H, Hanby M, Parkes LM, Gibson LM, Coutinho C, Emsley HC (2013). Prevalence and subtypes of radiological cerebrovascular disease in late-onset isolated seizures and epilepsy. Clin Neurol Neurosurg.

[21] Tartara E, Micalizzi E, Scanziani S, Ballante E, Paoletti M, Galimberti CA (2022). Late-onset focal epilepsy: electroclinical features and prognostic role of leukoaraiosis. Front Neurol.

[22] Johnson EL, Krauss GL, Lee AK, Schneider ALC, Kucharska-Newton AM, Huang J, Jack CR, Gottesman RF (2019). Association between white matter hyperintensities, cortical volumes, and late-onset epilepsy. Neurology.

[23] Jansen JFA, Vlooswijk MCG, Majoie HM, de Krom MCTFM, Aldenkamp AP, Hofman PAM, Backes WH (2008). White matter lesions in patients with localization-related epilepsy. Invest Radiol.

[24] De Reuck J, Goethals M, Claeys I, Van Maele G, De Clerck M (2006). EEG findings after a cerebral territorial infarct in patients who develop early- and late-onset seizures. Eur Neurol.

[25] Abraira L, Gramegna LL, Quintana M, Santamarina E, Salas-Puig J, Sarria S, Rovira A, Toledo M (2019). Cerebrovascular disease burden in late-onset non-lesional focal epilepsy. Seizure.

[26] Wahlund LO, Barkhof F, Fazekas F, Bronge L, Augustin M, Sjogren M, Wallin A, Ader H, Leys D, Pantoni L (2001). A new rating scale for age-related white matter changes applicable to MRI and CT. Stroke.

[27] Fazekas F, Chawluk JB, Alavi A, Hurtig HI, Zimmerman RA (1987). MR signal abnormalities at 1.5 T in Alzheimer's dementia and normal aging. AJR Am J Roentgenol.

[28] Shao IY, Power MC, Mosley T, Jack C, Gottesman RF, Chen LY, Norby FL, Soliman EZ, Alonso A (2019). Association of atrial fibrillation with white matter disease. Stroke.

[29] Calcetas AT, Thomas KR, Edmonds EC, Holmqvist SL, Edwards L, Bordyug M, Delano-Wood L, Brickman AM, Bondi MW, Bangen KJ (2022). Increased regional white matter hyperintensity volume in objectively-defined subtle cognitive decline and mild cognitive impairment. Neurobiol Aging.

[30] Kim KW, MacFall JR, Payne ME (2008). Classification of white matter lesions on magnetic resonance imaging in elderly persons. Biol Psychiatry.

[31] Gouw AA, Seewann A, van der Flier WM, Barkhof F, Rozemuller AM, Scheltens P, Geurts JJ (2011). Heterogeneity of small vessel disease: a systematic review of MRI and histopathology correlations. J Neurol Neurosurg Psychiatry.

[32] Fazekas F, Schmidt R, Scheltens P (1998). Pathophysiologic mechanisms in the development of age-related white matter changes of the brain. Dement Geriatr Cogn Disord.

[33] Izutsu N, Fujimoto Y, Yamada N, Kajikawa R, Yoshimura K, Nagashima M, Wakayama A, Yoshimine T (2018). Small hyperintensities in the area of the perforating arteries in patients with seizure. Eur Neurol.

[34] Mintzer S, Skidmore CT, Abidin CJ, Morales MC, Chervoneva I, Capuzzi DM, Sperling MR (2009). Effects of antiepileptic drugs on lipids, homocysteine, and C-reactive protein. Ann Neurol.

[35] Sarkis RA, Beers L, Farah E, Al-Akaidi M, Zhang Y, Locascio JJ, Properzi MJ, Schultz AP, Chhatwal JP, Johnson KA (2020). The neurophysiology and seizure outcomes of late onset unexplained epilepsy. Clin Neurophysiol.

[36] Tae WS, Ham BJ, Pyun SB, Kang SH, Kim BJ (2018). Current clinical applications of diffusion-tensor imaging in neurological disorders. J Clin Neurol.

