# Supplementary material for: Epileptogenicity of white matter lesions in cerebral small vessel disease: a systematic review and meta-analysis
Source: J Neurol. 2023 Jun 21;270(10):4890–902. doi: 10.1007/s00415-023-11828-6 (PMC10511556; doi:10.1007/s00415-023-11828-6)
Supplement: Supplementary file 1 — Supplementary file1 (PDF 456 KB) [file 415_2023_11828_MOESM1_ESM.pdf]

SUPPLEMENTAL MATERIAL – Journal of Neurology

Manuscript: Epileptogenicity of white matter lesions in cerebral small vessel disease: A systematic review and meta-analysis

Jakob I. Doerrfuss[1]; Jonas M. Hebel[1,*]; Martin Holtkamp[1,2,*]

[1] Charité – Universitätsmedizin Berlin, corporate member of Freie Universität Berlin and Humboldt-Universität zu Berlin, Department of Neurology with Experimental Neurology, Berlin, Germany

[2] Epilepsy-Center Berlin-Brandenburg, Institute for Diagnostics of Epilepsy, Berlin, Germany

[*] contributed equally

Corresponding Author:

Jakob I. Doerrfuss, Department of Neurology, Charité – Universitätsmedizin Berlin Campus Benjamin Franklin, Hindenburgdamm 30, 12203 Berlin, Germany

Email: jakob.doerrfuss@charite.de

Table E1 Search strategy

**MedLine database**<br><br>1 epilepsy/<br>2 epileps*.ti, ab.<br>3 seizures/<br>4 seizure*.ti,ab.<br>5 1 or 2 or 3 or 4<br>6 white matter/ab, dg, in, pa, pp [Abnormalities, Diagnostic Imaging, Injuries, Pathology, Physiopathology] 10112<br>7 white matter lesion*.ti,ab.<br>8 white matter disease.ti,ab.<br>9 white matter hyperintensit*.ti,ab.<br>10 white matter change*.ti,ab.<br>11 leukoencephalopathies/<br>12 leukoencephalopath*.ti,ab.<br>13 leukoaraiosis/<br>14 leukoaraiosis.ti,ab.<br>15 cerebral small vessel diseases/<br>16 cerebral small vessel disease*.ti,ab.<br>17 6 or 7 or 8 or 9 or 10 or 11 or 12 or 13 or 14 or 15 or 16<br>18 5 and 17<br>19 limit 18 to (yr="2003 - 2023" and english)<br>20 limit 19 to (case reports or comment or editorial or "review")<br>21 19 not 20
**Embase database**<br><br>1 epilepsy/<br>2 epileps*.ti,ab.<br>3 seizure/<br>4 seizure*.ti,ab.<br>5 1 or 2 or 3 or 4<br>6 white matter lesion*.ti,ab.<br>7 white matter disease.ti,ab.<br>8 white matter hyperintensit*.ti,ab.<br>9 white matter change*.ti,ab.<br>10 leukoencephalopathy/<br>11 leukoencephalopath*.ti,ab.<br>12 leukoaraiosis/<br>13 leukoaraiosis.ti,ab.<br>14 cerebral small vessel disease*.ti,ab.<br>15 6 or 7 or 8 or 9 or 10 or 11 or 12 or 13 or 14<br>16 5 and 15<br>17 limit 16 to (yr="2003 - 2023" and english)<br>18 limit 17 to ("preprints (unpublished, non-peer reviewed)" or (editorial or letter or "preprint (unpublished, non-peer reviewed)" or "review"))<br>19 17 not 18<br>20 case report.ti.<br>21 19 not 20

Table E2 - Newcastle – Ottawa Quality Assessment Scale (NOS) for all case-control studies included in this review

		Selection (seizures/epilepsy)				Comparability		Exposure (WML)			Quality score (0-9)
First author	Year	Case definition is adequate with independent validation	Consecutive or obviously representative series of cases	Community controls	Controls with no history of disease (endpoint)	Study controls for cardiovascula r risk factors	Study controls for additional factor	Ascertainment of exposure using secure record (eg surgical records) or structured interview where blind to case/control status	Same method of ascertainment for cases and controls	Non-response rate same for both groups	
**Abraira**	**2019**	☆	☆	-	☆	-	-	☆	☆	-	5
**De Reuck**	**2007**	☆	-	-	☆	-	-	☆	-	-	3
**Hanby**	**2015**	☆	☆	☆	☆	-	-	☆	☆	-	6
**Jansen**	**2008**	☆	-	☆	☆	-	-	☆	☆	-	5
**Johnson**	**2019**	-	☆	☆	☆	☆	☆	☆	☆	☆	8
**Mao**	**2016**	☆	☆	-	☆	☆	☆	☆	☆	☆	8
**Maxwell**	**2013**	☆	-	-	☆	-	-	☆	☆	-	4
**Stösser**	**2019**	☆	☆	-	☆	-	-	☆	☆	-	5
**Tartara**	**2022**	☆	☆	-	-	-	☆	☆	☆	☆	6
**Turon**	**2021**	☆	☆	-	☆	-	-	☆	☆	-	5
**Uslu**	**2021**	☆	☆	-	☆	-	☆	☆	☆	☆	7

WML white matter lesions. The scale ranges from 0 to 9 points (each star represents one point) with a higher score indicating a superior quality. A score of ≥ 7 indicates a study of high methodical quality.

Table E3 - In patients with first seizure or epilepsy, do the amount and localization of WML of presumed vascular origin differ in comparison to people who never had a seizure?

Author	Year	Properties of epilepsy group	WML load epilepsy group	WML load control group		Main results regarding WML load
Abraira	2019	- patients with epilepsy, onset ≥ 60 years<br>- MRI with no epileptogenic lesions<br>- psychogenic non-epileptic seizures and neurodegenerative diseases excluded	Fazekas 0: 15 (45.5%)<br>Fazekas 1: 15 (45.5%)<br>Fazekas 2: 3 (9.1%)<br>Fazekas 3: 0 (0)	Control group I<br>Fazekas 0: 11 (26.8%)<br>Fazekas 1: 12 (29.3%)<br>Fazekas 2: 17 (41.5%)<br>Fazekas 3: 1 (2.4%)	Control group II<br>Fazekas 0: 15 (57.7%)<br>Fazekas 1: 9 (34.6%)<br>Fazekas 2: 1 (3.8%)<br>Fazekas 3: 1 (3.8%)	- WML load lower in patients with epilepsy vs. control group I, p = 0.004<br>- WML load not statistically significantly different in epilepsy vs. control group II, p=0.593
De Reuck	2007	- patients with at least one seizure with history of prior lacunar stroke<br>- no patients with history of head trauma, birth complications, drug abuse or family history of epilepsy<br>- age of participants not reported	Degree of white matter changes (not further specified)<br>0.59	Degree of white matter changes (not further specified)<br>0.64		- no statistically significant difference in degree of white matter changes in seizure/epilepsy group vs. control group, p=0.524.
Hanby	2015	- patients with epilepsy, onset > 50 years<br>- MRI with no epileptogenic lesions<br>- no patients with history of stroke or transient ischemic attack or severe cognitive dysfunction	White matter hyperintensity volume: 1,339 mm^3 (+/- 1,408)	White matter hyperintensity volume: 514 mm^3 (+/- 481)		- white matter hyperintensity volume higher in epilepsy group vs. control group, p=0.047
Jansen	2008	- patients with focal epilepsy of unknown etiology, no age restrictions<br>- no patients with structural epilepsy, history of status epilepticus, progressive neurological disorder, cognitive decline	- WML volume: 160 mm^3 (+/- 577)<br>- prevalence of subcortical white matter lesions: 46%	- WML volume: 203 mm^3 (+/- 350)<br>- prevalence of subcortical white matter lesions: 63%		- no statistically significant difference in subcortical WML in epilepsy group vs. control group, p=0.7
Johnson	2019	- participants of a community-based study on atherosclerosis risk with ≥ 2 seizures occurring at an age of ≥ 60 years according to hospitalization records and Medicare claims<br>- MRI was performed *before* onset of first seizure<br>- no patients with multiple sclerosis, brain tumors, brain surgery or brain radiation	- WML volume: 21,800 mm^3 (+/- 17,500)<br>- median white matter grade 1 (quartiles 1-2)	- WML volume: 17,500 mm^3 (+/- 16,500)<br>- median white matter grade 1 (quartiles 1-2)		- no statistically significant difference in WML volume in patients who developed epilepsy later in life vs. control group, p=0.163<br>- WML volume not associated with later development of epilepsy, OR 1.33, 95%CI 0.85-2.08<br>- white matter grade independently associated with later development of epilepsy, HR 1.27, 95%CI 1.06–1.54
Mao	2016	- patients with epilepsy, no age restrictions<br>- new onset epilepsy and chronic epilepsy analyzed separately<br>- no patients with history of stroke, transient ischemic attack, multiple sclerosis, signs of “bi-hemispheric brain injury” (trauma, tumor, surgical procedures)	- prevalence of white matter lesions: 6.4%<br>- ARWMC 0: 73 (33.6%)<br>ARWMC 1-2: 80 (36.9%)<br>ARWMC 3-4: 38 (17.5%)<br>ARWMC 5-8: 23 (10.6%)<br>ARWMC 9-12: 3 (1.4%)	- prevalence of white matter lesions 43.5%<br>- ARWMC 0: 13 (56.5%)<br>ARWMC 1-2: 8 (34.8%)<br>ARWMC 3-4: 1 (4.3%)<br>ARWMC 5-8: 0 (0%)<br>ARWMC 9-12: 1 (4.3 %)		- significantly higher WML scores in patients with epilepsy vs. control group, IRR 1.93, 95%CI 1.13-3.30 (adjusted for age)

Author	Year	Properties of epilepsy group	WML load epilepsy group	WML load control group	Main results regarding WML load
Maxwell	2013	- patients with one unprovoked seizure or epilepsy, onset > 60 years,	- prevalence of WML: 58.1%<br>- Fazekas 0: 61 (58.1%)<br>Fazekas 1: 28 (26.7%)<br>Fazekas 2: 11 (10.5%)<br>Fazekas 3: 5 (4.8%)	- prevalence of WML 29.5%<br>- Fazekas 0: 15 (45.5%)<br>Fazekas 1: 15 (45.5%)<br>Fazekas 2: 3 (9.1%)<br>Fazekas 3: 0 (0)	- small vessel disease (WML, leukoaraiosis, lacunar infarcts, and enlarged perivascular spaces) statistically significant more frequent in epilepsy group vs. control group<br>- Fazekas score non-significantly higher in epilepsy group vs. control group, p=0.06, 0.88, 0.05, 0.06 for Fazekas 0,1,2,3 respectively
Stösser	2019	- patients with first focal impaired awareness seizure; ≥ 60 years<br>- no patients with epilepsy, epileptogenic lesions on MRI and moderate to severe cognitive impairment	- global Fazekas 0: 4 (3.4%)<br>global Fazekas 1: 34 (28.8%)<br>global Fazekas 2: 34 (28.8%)<br>global Fazekas 3: 46 (39.0%)<br>- prevalence of juxtacortical lesions: 80.5%	- global Fazekas 0: 9 (7.6%)<br>global Fazekas 1: 68 (57.6%)<br>global Fazekas 2: 27 (22.9%)<br>global Fazekas 3: 14 (11.9%)<br>- prevalence of juxtacortical lesions: 22.0%	- significantly higher WML scores in patients with seizure vs. control group, p<0.001<br>- juxtacortical lesions more common in seizure group vs. control group (80.5% vs 22.0%, p < 0.001)
Turon	2021	- patients with ≥ 1 unprovoked seizure *and* incidental diagnosis of CSVD (subcortical microbleeds, Fazekas score ≥ 1 or ≥ 1 lacunar infarct), aged ≥ 60 years<br>- no patients with neurodegenerative diseases, epileptogenic lesion, autoimmune diseases, or psychogenic nonepileptic seizures	- Fazekas 0-1: 20 (74.1%)<br>Fazekas 3-4: 7 (25.9%)	- Fazekas 0-1: 25 (92.6%)<br>Fazekas 3-4: 2 (7.4%)	- patients with epilepsy had non-significantly more often Fazekas score >2, p=0.068)<br>- CSVD burden score (including lacunar infarcts, cerebral microbleeds, Fazekas score > 2, enlarged perivascular spaces) higher in epilepsy vs. non-epilepsy group, p = 0.012
Uslu	2021	- patients with epilepsy ≥ 18 years old<br>- no patients with unclassified epilepsy, acute symptomatic seizures or isolated unprovoked seizure<br>- no patients with vascular risk factors (hypertension, diabetes mellitus, cardiac diseases), no neurodegenerative diseases or other central nervous system diseases, or cerebral lesions other than WMH	- prevalence of WML: 27.7%	- prevalence of WML: 14.6%	- no statistically significant difference in presence of WML between both groups p=0.126<br>- healthy controls: WMH only in frontal lobes, epilepsy: WMH in frontal, parietal, temporal and occipital localization (only descriptive)

WMH white matter hyperintensity, WML white matter lesions, TIA transient ischemic attack, OR odds ratio, HR hazards ratio, IRR incidence rate ratio, 95%CI 95% confidence interval

Table E4 - In patients with a first seizure or epilepsy, is the presence of WML of presumed vascular origin associated with an increased risk of recurrent seizures as compared to patients without WML?

Author	year	Definition of CSVD	Patient collective	Results regarding seizure frequency
Uslu	2021	CSVD defined as Fazekas ≥ 1	- patients with epilepsy, ≥ 18 years old<br>- no patients with unclassified epilepsy, acute symptomatic seizures or isolated unprovoked seizure<br>- no patients with vascular risk factors (hypertension, diabetes mellitus, cardiac diseases), no neurodegenerative diseases or other central nervous system diseases, or cerebral lesions other than WMH	- presence of WML not associated with frequency of seizures (p=0.444); “frequency of seizures” not defined
Tartara	2022	CSVD defined as Fazekas ≥ 1	- patients with newly diagnosed epilepsy, ≥ 60 years old<br>- epilepsy of unknown cause or structural cause<br>- no patients with dementia, psychogenic seizures, rapidly growing CNS tumors	- absence of WML associated with statistically significant higher probability of achieving seizure freedom, p=0.012<br>- presence of WML independently associated with higher risk of seizure recurrence, OR 1.8, 95%CI 1.042-3.110)<br>- the severity of WML did not affect the outcome regarding seizure recurrence

CSVD cerebral small vessel disease
